# Loop Diuretics Have Anxiolytic Effects in Rat Models of Conditioned Anxiety

**DOI:** 10.1371/journal.pone.0035417

**Published:** 2012-04-13

**Authors:** Andrew D. Krystal, Janice Sutherland, Daryl W. Hochman

**Affiliations:** 1 Department of Psychiatry and Behavioral Sciences, Duke University Medical Center, Durham, North Carolina, United States of America; 2 Department of Neuroscience, University of Lethbridge, and NeuroInvestigations Inc., Lethbridge, Alberta, Canada; 3 Departments of Surgery (Surgical Sciences) and Pharmacology and Cancer Biology, Duke University Medical Center, Durham, North Carolina, United States of America; Radboud University, Netherlands

## Abstract

A number of antiepileptic medications that modulate GABA_A_ mediated synaptic transmission are anxiolytic. The loop diuretics furosemide (Lasix) and bumetanide (Bumex) are thought to have antiepileptic properties. These drugs also modulate GABA_A_ mediated signalling through their antagonism of cation-chloride cotransporters. Given that loop diuretics may act as antiepileptic drugs that modulate GABAergic signalling, we sought to investigate whether they also mediate anxiolytic effects. Here we report the first investigation of the anxiolytic effects of these drugs in rat models of anxiety. Furosemide and bumetanide were tested in adult rats for their anxiolytic effects using four standard anxiety models: 1) contextual fear conditioning; 2) fear-potentiated startle; 3) elevated plus maze, and 4) open-field test. Furosemide and bumetanide significantly reduced conditioned anxiety in the contextual fear-conditioning and fear-potentiated startle models. At the tested doses, neither compound had significant anxiolytic effects on unconditioned anxiety in the elevated plus maze and open-field test models. These observations suggest that loop diuretics elicit significant anxiolytic effects in rat models of conditioned anxiety. Since loop diuretics are antagonists of the NKCC1 and KCC2 cotransporters, these results implicate the cation-chloride cotransport system as possible molecular mechanism involved in anxiety, and as novel pharmacological target for the development of anxiolytics. In view of these findings, and since furosemide and bumetanide are safe and well tolerated drugs, the clinical potential of loop diuretics for treating some types of anxiety disorders deserves further investigation.

## Introduction

Anxiety disorders are the most prevalent class of psychiatric conditions, affecting approximately 18% of adults [1]–[3]. These disorders include Panic Disorder (PD), Social Anxiety Disorder (SAD), Obsessive Compulsive Disorder (OCD), Posttraumatic Stress Disorder (PTSD), Generalized Anxiety Disorder (GAD), and Specific Phobia [4]. Medications currently used for treating these disorders include tricyclic antidepressants, selective serotonin reuptake inhibitors (SSRIs), serotonin norepinephrine reuptake inhibitors (SNRIs), benzodiazepines, anticonvulsants, and monoamine oxidase inhibitors. However, 20%–40% of anxiety patients remain non-responders to all available therapies [5]. Additionally, many of the anxiolytic medications can elicit central nervous system (CNS) side-effects that patients find difficult to tolerate [5], [6]. There is a need for new pharmacotherapeutic approaches to treat anxiety with greater efficacy and fewer side effects.


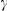
-aminobutyric acid (GABA)is the primary inhibitory neurotransmitter in the CNS. The downregulation of GABA_A_ inhibition in the brain has been hypothesized to contribute to pathophysiological anxiety [7]. Antiepileptic drugs that enhance GABA_A_ signaling often possess anxiolytic properties and are commonly prescribed to treat anxiety. These drugs include pregabalin for GAD, pregabalin and gabapentin for SAD, and a number of benzodiazepines for GAD, SAD, and panic disorder [8]. The loop diuretics furosemide (Lasix) and bumetanide (Bumex) are also thought to be GABA_A_ modulators with antiepileptic properties [9]–[12]. These drugs have attracted some interest from epilepsy researchers because of their antiepileptic effects over a wide variety of experimental seizure models [9], [11], [13], [14], and several clinical findings suggesting they can suppress seizures in patients with medically intractable epilepsy [15], [16].

Loop diuretics are thought to affect GABA_A_ dependent signaling in the brain through their antagonism of cation-chloride cotransport, which is a distinctly different mechanism of action from all other known pharmacological GABA_A_ modulators [17]. Specifically, furosemide and bumetanide antagonize the Na^+^-K^+^-2Cl^−^ (NKCC1) cotransporter that is present on both neurons and glial cells, and the neuron-specific K^+^-Cl^−^ (KCC2) cotransporter [10], [11], [18]–[20]. NKCC1 normally transports chloride from the extracellular to intracellular spaces, and KCC2 transports chloride from intracellular to extracellular spaces. Although furosemide and bumetanide are thought to antagonize both cotransporters, they both have significantly greater affinity for NKCC1 over KCC2 [10]. Hyperpolarizing inhibitory postsynaptic potentials in neurons are generated by the influx of anions (HCO_3_^−^ and Cl^−^) down their electrochemical gradients [21]. Since GABA_A_ receptor-mediated current is determined, in part, by the difference between the equilibrium potential for Cl^−^ and the neuronal membrane potential [22], preferential antagonism of NKCC1 with a loop diuretic would be expected to cause a hyperpolarizing shift in the GABA reversal potential, enhancing GABA_A_ synaptic signalling. This effect can be particularly important in view of recent work showing the dominant role that NKCC1 plays at the axon initial segment of principal neurons [23], [24].

Given that loop diuretics possibly act as antiepileptic agents that enhance GABA_A_ inhibition, we sought to investigate whether they also mediate anxiolytic effects. Towards that end, we tested the anxiolytic effects of furosemide (100 mg/kg I.V.) and bumetanide (70 mg/kg I.V.), on four standard rat anxiety models: 1) Contextual Fear-Conditioning which measures fear, in terms of freezing, linked to a context where footshock occurred [25], [26]; 2) Fear-Potentiated Startle which measures conditioned fear in terms of the increase in the startle reflex elicited by sudden noise in the presence of a cue that was previously paired with footshock [27], [28]; 3) Elevated Plus Maze which assesses unconditioned fear in terms of the degree to which rats explore regions that normally elicit their fear of heights and lighted un-enclosed spaces [29]; and 4) Open-Field Test, which assesses unconditioned fear in terms of the degree to which rats' innate fear of a novel and well-lit open field impedes their desire to explore new environments [30]. The doses of the furosemide and bumetanide were chosen to be similar to those previously shown to suppress kainic acid induced seizures in adult rats [9], [31].

## Results

### Contextual Fear-Conditioning

**Figure 1:**
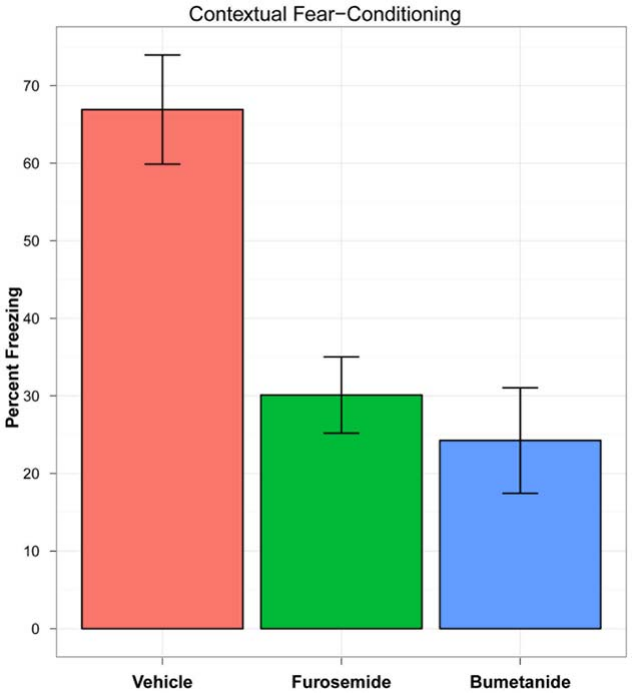
Contextual Fear-Conditioning Results. Percentage of time during the contextual fear-conditioning test period during which rats were freezing, following intravenous injections of vehicle (N = 8), bumetanide (N = 8), and furosemide (N = 8). Note: Error bars indicate standard errors.

The rats treated with bumetanide (N = 8) and furosemide (N = 8) spent a significantly smaller percentage of the test period freezing compared to the rats treated with vehicle alone (N = 8) (vehicle mean = 66.914 [SE = 7.04]; bumetanide mean = 24.3 [SE = 6.80]; furosemide mean = 30.12 [SE = 4.91]) (df = 2; F = 13.382; p<0.0001) (see Figure 1).

### Fear-Potentiated Startle

**Figure 2:**
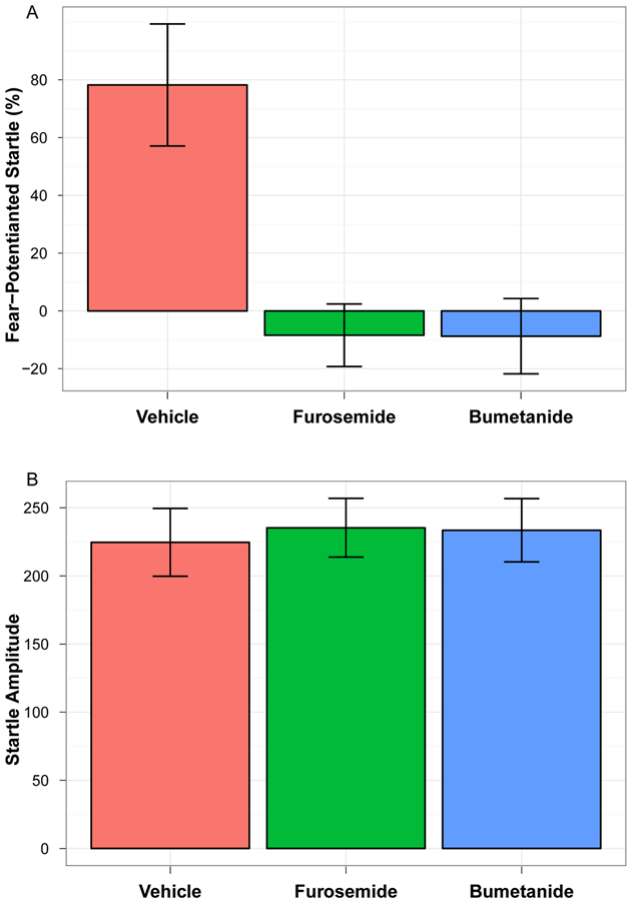
Fear-Potentiated Startle Test Results. Startle amplitudes for rats receiving intravenous injections of vehicle (N = 7), rats receiving furosemide (N = 8), and rats receiving bumetanide (N = 8). (A) Percent amount of fear-potentiated startle, and (B) amplitude of startle to the noise alone. Note: Error bars indicate standard errors.

The rats treated with bumetanide (N = 8) and furosemide (N = 7) had significantly less increase in startle amplitude with the shock-conditioned stimulus than rats treated with vehicle alone (N = 8) (vehicle mean = 78.22 [SE = 21.10]; bumetanide mean = −8.75 [SE = 13.03]; furosemide mean = −8.42 [SE = 10.82]) (df = 2; F = 9.99; p<0.001) (see Figure 2).

### Elevated Plus Maze

No significant differences were seen for rats treated with bumetanide (N = 8) and furosemide (N = 8) compared with those treated with vehicle alone (N = 8) on entries into closed arms, time spent in the open, and trips to at least the midpoint of open arms (see Table 1).

**Table 1:** Elevated Plus Maze Results.

	Vehicle Mean (SE)	Furosemide Mean (SE)	Bumetanide Mean (SE)
**Closed Arm Entries**	6.13 (0.69)	5.25 (0.59)	5.13 (0.40)
**Time Spent in Open**	58.13 (8.65)	56.25 (6.51)	68.75 (11.30)
**Trips Down Open Arms**	0.50 (0.27)	0.63 (0.32)	0.88 (0.35)

### Open-Field Test

No significant differences were seen for rats treated with bumetanide (N = 8) and furosemide (N = 8) compared with those treated with vehicle alone (N = 8) on the total distance travelled in the open-field, total time spent moving in the open-field, number of rears, time spent rearing, distance travelled within the margin of the field, time spent within the margin of the field, distance travelled within the center of the open-field, and time spent within the center of the open-field (see Table 2).

**Table 2:** Open-Field Test Results.

	Vehicle Mean (SE)	Furosemide Mean (SE)	Bumetanide Mean (SE)
**Total Distance Travelled**	1506.25 (341.20)	1550.88 (290.81)	2411.88 (359.52)
**Time Spent Moving**	138.90 (25.59)	150.53 (21.34)	206.65 (28.00)
**Number of Rears**	31.50 (5.37)	40.13 (6.15)	49.13 (7.95)
**Time Spent Rearing**	129.05 (29.34)	117.49 (26.31)	188.39 (37.26)
**Distance Travelled Within Margin**	926.50 (232.06)	970.5 (197.22)	1410.50 (179.20)
**Time Spent Within Margin**	468.88 (35.15)	423.48 (27.35)	447.84 (28.62)
**Distance Travelled Within Center**	579.63 (134.06)	580.25 (113.73)	1001.75 (202.74)
**Time Spent Within Center**	131.13 (35.15)	176.53 (27.35)	152.16 (28.62)

## Discussion

The findings of this study suggest that, at the doses tested, furosemide and bumetanide have significant anxiolytic effects in the conditioned models of anxiety (contextual fear-conditioning, fear-potentiated startle) but not in the unconditioned models of anxiety (elevated plus maze, open-field test). Since only single doses furosemide and bumetanide were tested in each of the models, we can not exclude the possibility that these drugs would also show anxiolytic effects in the unconditioned models of anxiety at higher doses. In view of this possibility, our data here only supports the conclusion that these drugs are more potently anxiolytic in conditioned models of anxiety than they are in unconditioned models. Indeed, there does appear to be a small effect of bumetanide in the open-field test, although it is not a statistically significant effect. Further experiments, using higher doses of furosemide and bumetanide, would be required to determine whether or not these compounds are specifically anxiolytic only for conditioned anxiety in rat models.

Bumetanide and furosemide both have greater affinity for NKCC1 over KCC2, and bumetanide has a very low affinity for KCC2 [10]. In view of this consideration, it seems more probable that these drugs mediate their anxiolytic effects through their antagonism of NKCC1 rather than KCC2, since it might otherwise be expected that bumetanide would be ineffective. However, dose response studies of the efficacies of furosemide and bumetanide in these anxiety models would be required to quantify their relative potencies for mediating anxiolytic effects, and thus necessary for providing more conclusive evidence of the specific cation-chloride cotransporter involved. In addition to their antagonism of NKCC1 and KCC2, it is always possible that furosemide and bumetanide might affect other ion channels and transporters in brain that could play a role in their anxiolytic effects. For example, high concentrations of these drugs in other tissues have been found to inhibit Cl^−^/HCO_3_^−^-exchange and some types of Cl^−^ channels [32], [33]. However, the currently available data suggests that these drugs have far greater affinity for NKCC1 and KCC2 over these other putative targets, making such alternative mechanisms less likely [10].

The rationale motivating the work here was that since loop diuretics are possibly both antiepileptic and GABA_A_ modulating, then they might also be anxiolytic similar to other GABA_A_ modulating antiepileptic drugs. However, the actual mechanisms through which chloride-cotransport antagonism might mediate the anxiolytic effects observed here remain unknown. Indeed, one possibility is that these medications increase the transmembrane chloride gradients of neurons, which would increase hyperpolarization occurring with GABA_A_ receptor activation-related chloride channel opening [11], [13]. Increasing GABA_A_ mediated inhibition is also thought to be the mechanism through which benzodiazepines mediate their anxiolytic effects [8]. However, antagonism of the cation-chloride cotransporters with loop diuretics also mediate a number of other important CNS effects, such as changes in cell volumes and extracellular ion concentrations, that can significantly influence the synchronization of neuronal firing activity [16], [31], [34]. One possible concern regarding the interpretation of our results is that the anxiolytic effects observed here might have been the result from some systemic effect of diureses, rather than from a CNS-specific pharmacological action of the loop diuretics on neuronal and glial chloride-cotransporters. We feel this possibility is an unlikely explanation for our observations, since then, one might have expected to observe a similar anxiolytic effect in all four of the models tested. This possibility could be more definitively tested, for example, by repeating the experiments described here with other diuretics that have no affinity for the cation-chloride cotransporters. Further work will be needed to determine the specific mechanisms by which chloride-cotransport antagonism leads to the observed anxiolytic effect.

The doses of furosemide and bumetanide used here to elicit anxiolytic effects in rats are large by comparison to their standard doses when used clinically as diuretics for humans. In order to translate the doses used here in the rats into ones that might be anxiolytic in humans, it is important to note that loop diuretics are metabolized much more rapidly and efficiently in rats than they are in the humans [35], [36]. Furosemide is 230 times less potently diuretic in rats than in humans, with a half-life of 11 minutes in rats compared with approximately 2 hours in humans [35]. Bumetanide is proportionally even less potent than furosemide between rats and humans [36]. This suggests that, if loop diuretics are indeed anxiolytic in humans, then much smaller doses than used here would be required to achieve a therapeutic effect. Such a species-specific difference in the therapeutic effects of loop diuretics has been observed in studies of their antiepileptic properties in rats and humans. Large doses, similar to the ones used here, are required to suppress seizure activity in rats [9], [31], whereas standard clinical doses are sufficient to reduce seizure activity in humans [15], [16]. This suggests that, if loop diuretics are indeed anxiolytic in humans, then standard clinical doses of bumetanide (0.5–1.0 mg/dose) and furosemide (20–40 mg/dose) might be sufficient to elicit anxiolytic effects in the humans. In humans, oral doses of bumetanide and furosemide are quickly absorbed and have good bioavailability [37].

Further work will also be needed to determine whether NKCC1 antagonists have anxiolytic effects in humans and, if so, which anxiety disorders are improved by these agents. Although the results of this study suggest that NKCC1 antagonism might improve disorders where conditioned anxiety plays an important role, such as PTSD, and not disorders marked by unconditioned anxiety, such as GAD, the available literature suggests that this may not be the case (see Table 3). For example, benzodiazepines, which reliably demonstrate therapeutic effects in the tests of conditioned anxiety (contextual fear conditioning and fear-potentiated startle), do not have a therapeutic effect on the specific symptoms of PTSD, though they improve non-specific anxiety in PTSD patients [8]. The effects of NKCC1 antagonists on the four anxiety tests studied are unlike those of any of the major classes of medications with anxiolytic effects in humans (benzodiazepines, tricyclic antidepressants, SSRIs, SNRIs, and 5HT1A agonists), in that they appear to be specific to the conditioned models (see Table 3). Nonetheless, positive effects on these tests reliably predict some type of therapeutic anxiolytic effects in humans [38]. As such, this study provides some evidence for a new mechanism of anxiety and class of medications with potential for treating anxiety disorders.

**Table 3:** Medications with Established Human Anxiolytic Effects.

Medication Type	Contextual Fear-Conditioning	Fear-Potentiated Startle	Elevated Plus Maze	Open-Field Test	Efficacy in Human PD	Efficacy in Human GAD	Efficacy in Human PTSD	Efficacy in Human SAD
Loop Diuretics	+	+	–	–	?	?	?	?
Benzodiazepines	+	+	+	+	+/–	+	–	+
Tricyclic Antidepressants	+	–	–	?	+	+	+/–	?
SSRIs/SNRIs	+	–	+/–	–	+	+	+*	+
5HT1A Agonists	?	+/–	+/–	+	–	+	–	–

+ At least one placebo-controlled study with the preponderance demonstrating an anxiolytic effect; -At least one placebo-controlled study with the preponderance not finding an anxiolytic effect; +/− At least one placebo-controlled study and the findings are equivocal and/or there is no clear preponderance of positive or negative results; ? No placebo-controlled studies have been carried out. PD = Panic Disorder; GAD = Generalized Anxiety Disorder; PTSD = Post-Traumatic Stress Disorder; and SAD = Social Anxiety Disorder. *Although there are a number of positive studies with SSRIs, the Institute of Medicine concluded that there was insufficient evidence to support the efficacy of SSRIs in PTSD due to moderate effect sizes [8], [27], [28], [30], [35], [38], [46]–[52].

If chloride-cotransporter antagonists are effective in humans as predicted by these animal models, they would be mediating their therapeutic effects through a unique mechanism and molecular target, and potentially have certain advantages over existing agents in that they would be the only agents with immediate onset of action (unlike SSRIs/SNRIs), and without the sedation, cognitive impairment, and the abuse potential of benzodiazepines [6], [39]. Furosemide has been safely used clinically since 1966 to treat millions of patients for hypertension, edema, and heart failure, and was ranked in 2008 as being the 17th most frequently prescribed drug [40]. The need for improved anxiety therapeutics suggests it may be worthwhile to carry out studies in humans with anxiety disorders to determine the clinical utility of loop diuretics [5], [6].

## Materials and Methods

### Ethics Statement

All procedures were performed in accordance with the University of Lethbridge Animal Care Committee guidelines, which follow the standards set by the Canadian Council on Animal Care. This study was conducted under a protocol titled: “Assessment of the Therapeutics Potential of Bumetanide and Furosemide in Treatment of Addictions, PTSD, and Anxiety in Rats, approved by UNIVERSITY OF LETHBRIDGE Animal Welfare Committee (AWC). Protocol #0513.

### Animal Handling and Drug Delivery

Ninety-six male, adult (3–4 months old) Long-Evans rats, housed in the University of Lethbridge vivarium, were used for these studies. Rat housing consisted of Plexiglas cages with sawdust bedding shared with two or three individuals. The colony room was temperature-controlled (20–21°C) with a 12 h light/12 h dark cycle, beginning each day at 07:00. Food and water were provided ad libitum. Seventy-two hours prior to the experiment, rats were anaesthetized with isoflurane, and a cannula was implanted into the right external jugular vein of each rat for the purpose of administration of drugs [41]. Rats were thereafter kept in independent cages, and the cannulas were flushed daily to ensure patency. Bumetanide and furosemide were dissolved in DMSO (vehicle), and all drugs were administered I.V. via a cannulated jugular vein. Test drugs were administered 30 min prior to testing. All behavioural testing was conducted during the light cycle (7:00 am–7:00 pm). Testing occurred between the hours of 9:00 am and 3:00 pm. Different, randomly selected rats were used for each group (i.e. no rat was retested in more than one group). All testing was done under ambient room light.

### Contextual Fear-Conditioning

Contextual Fear-Conditioning Contextual Fear-Conditioning, following a previously described standard protocol, was performed on 24 rats [42]. The testing chamber consisted of a rectangular box (40 cm×56 cm×28 cm) with a stainless steel rod floor. All aspects of the timing of events were under microcomputer control (MedPC, MedAssociates Inc, Vermont, USA). Measurement of freezing was accomplished through an overhead video camera connected to a microcomputer and was automatically scored using a specialty piece of software, FreezeFrame. In Phase 1, rats were placed individually into the chambers for 5 minutes. Phase 2 occurred 24 hr later, when again rats were placed individually into the same chambers, they received an immediate (within 3 s of being placed into the chamber) foot shock (1 mA for 2 s). Thirty seconds later they were removed from the chambers. During phase 3, 24 hr later, the rats were returned to the chambers for 5 min. This session was video recorded and the amount of time spent freezing was assessed using FreezeFrame software. Freezing was defined as the total lack of body movement except for movement related to respiration. The percentage time spent freezing during each minute was entered into Excel spreadsheets and was analyzed using SPSS statistical software. One-way analysis of variance (ANOVA) was used to evaluate treatment effects.

### Fear-Potentiated Startle

A Fear-Potentiated Startle protocol, following a previously described protocol, was used to test 23 rats [43]. Animals were trained and tested in four identical stabilimeter devices (Med-Associates). Each rat was placed in a small Plexiglas cylinder. The floor of each stabilimeter consisted of four 6-mm-diameter stainless steel bars spaced 18 mm apart through which shock can be delivered. Cylinder movements result in displacement of an accelerometer where the resultant voltage is proportional to the velocity of the cage displacement. Startle amplitude was defined as the maximum accelerometer voltage that occurs during the first 0.25 sec after the startle stimulus was delivered. The analog output of the accelerometer was amplified, digitized on a scale of 0–4096 units and stored on a microcomputer. Each stabilimeter was enclosed in a ventilated, light-, and sound-attenuating box. All sound level measurements were made with a Precision Sound Level Meter. The noise of a ventilating fan attached to a sidewall of each wooden box produces an overall background noise level of 64 dB. The startle stimulus was a 50 ms burst of white noise (5 ms rise–decay time) generated by a white noise generator. The visual conditioned stimulus was the illumination of a light bulb adjacent to the white noise source. The unconditioned stimulus was a 0.6 mA foot shock with duration of 0.5 s, generated by four constant-current shockers located outside the chamber. The presentation and sequencing of all stimuli were controlled by computer. FPS procedures consist of 5 days of testing; during days 1 and 2 baseline startle responses were collected, days 3 and 4 light/shock pairings were delivered, day 5 testing for fear potentiated startle was conducted. Animals received treatment with compound or vehicle on days 3, 4, and 5.

#### Matching

On days 1 and 2 rats were placed individually into the Plexiglas cylinders and 3 min later presented with 30 startle stimuli at a 30 sec interstimulus interval. An intensity of 105 dB was used. The mean startle amplitude across the 30 startle stimuli on the second day was used to assign rats into treatment groups with similar means.

#### Training

On days 3 and 4, rats were placed individually into the Plexiglas cylinders. During the first 3 min in the chamber the rats were allowed to acclimate then 10 CS-shock pairings were delivered. The shock was delivered during the last 0.5 sec of the 3.7 sec CSs at an average intertrial interval of 4 min (range, 3–5 min).

#### Testing

On the 5th day, rats were placed in the same startle boxes where they were trained and after 3 min acclimation were presented with 18 startle-eliciting stimuli (all at 105 dB). These initial startle stimuli were used to again habituate the rats to the acoustic startle stimuli. Thirty seconds after the last of these stimuli, each animal receives 60 startle stimuli with half of the stimuli presented alone (startle alone trials) and the other half presented 3.2 sec after the onset of the 3.7 sec CS (CS-startle trials). All startle stimuli were presented at a mean 30 sec interstimulus interval, randomly varying between 20 and 40 sec. Data were entered into Excel spreadsheets and SPSS for data analysis. Independent sample t-tests are used to compare each treatment groups.

### Elevated Plus Maze

An Elevated Pluse Maze protocol was used to test 24 rats [44]. The elevated plus maze consisted of two opposing open arms, 50×10 cm, crossed with two opposing enclosed arms of the same dimensions but with walls 40 cm high. Each of the four arms was connected to one side of a central square (10×10 cm) giving the apparatus a plus-sign appearance. The maze was elevated 50 cm above the floor in a normally illuminated room. The rats were placed individually on the central square of the plus maze facing an enclosed arm. The entire 3-min session was video taped and later scored. The time spent and the number of entries into the open and closed arms, and the number of trips made to at least the mid point down the open arms were recorded. An arm entry was defined as placement of all four paws onto the surface of the arm. The treatment groups were compared on time in the open, closed arm entries, and trips to at least the midpoint down open arms with ANOVA.

### Open-Field Test

Twenty-four rats underwent a standard Open-Field Test, following a previously published protocol [45]. The open field consisted of a latex-painted, circular wooden table, 155 cm in diameter, raised 64 cm above the floor. To avoid scented residue confounds, a ball bearing base allowed the table to be rotated between trials. The table was also wiped down with soapy water after each trial. The table was located in a large room, painted white, which contained several visual cues including light switches, electrical outlets, a paper towel dispenser, a door, and two posters. A ceiling-mounted, wide-angle lens video camera recorded each trial in Standard Play format onto Mini Digital Videocassettes for later analysis. During open field sessions, each rat was brought into the testing room from its home cage and placed in the center of the open field, away from the edges of the table to avoid the influence of point of entry on location preference. The experimenter then left the room and initiated video recording. Sessions lasted 10 min, after which the rat was removed from the table and returned to its home cage. To examine the amount of time the rats spend in the various quadrants of the table, an AccuTrak software program transformed the position of the rat on the Table into a series of Cartesian coordinates sampled at a rate of 30 Hz. Custom software divided the open field Table into four quadrants, and used the AccuTrak output coordinates to determine the amount of time the rat spent in each quadrant over the course of the trial. Time accrued within these quadrants was measured as a sum for the entire 30-min trial, and as experimenter-assigned 3-min bins, for a total of 10 time-bins. Rearing, distance travelled, and time spent in field regions were compared across treatment groups with ANOVA.
